# Evaluation of the American Society of Gastrointestinal Endoscopy 2019 and the European Society of Gastrointestinal Endoscopy guidelines' performances for choledocholithiasis prediction in clinically suspected patients: A retrospective cohort study

**DOI:** 10.1002/jgh3.12773

**Published:** 2022-05-25

**Authors:** Suppadech Tunruttanakul, Borirak Chareonsil, Kotchakorn Verasmith, Jayanton Patumanond, Chatchai Mingmalairak

**Affiliations:** ^1^ Department of Surgery Sawanpracharak Hospital Nakhon Sawan Thailand; ^2^ Department of Radiology Sawanpracharak Hospital Nakhon Sawan Thailand; ^3^ Center for Clinical Epidemiology and Clinical Statistics, Faculty of Medicine Chiang Mai University Chiang Mai Thailand; ^4^ Department of Surgery, Faculty of Medicine Thammasat University Pathum Thani Thailand

**Keywords:** ability, choledocholithiasis, guideline, prevalence

## Abstract

**Background and Aim:**

The American Society of Gastrointestinal Endoscopy (ASGE) and the European Society of Gastrointestinal Endoscopy (ESGE) have published guidelines for choledocholithiasis. However, the guidelines were formulated using data from a large number of patients with no to low risk of common bile duct (CBD) stones. This study aimed to assess the guidelines' predictive performance in a population with a high frequency of stones.

**Methods:**

Data for three choledocholithiasis standard reference tests were retrospectively reviewed from January 2019 to June 2021. Clinical parameters were used to categorize patients into risk groups according to the guidelines, and then the guidelines' predictive abilities were calculated.

**Results:**

Among 1185 patients, 521 were included. The stone prevalence was 61.0% (*n* = 318). Twelve (2.3%), 146 (28.0%), and 363 (69.7%) patients were classified into low‐, intermediate‐, and high‐risk groups according to the ASGE guidelines, and 30 (5.8%), 149 (28.6%), and 342 (65.6%) according to the ESGE guidelines. Focusing on the high‐risk group, the ASGE guidelines had a positive predictive value of 73.6 and a positive likelihood ratio of 1.78. The ESGE guidelines had a positive predictive value of 73.7 and positive likelihood ratio of 1.79. Both guidelines had equivalent areas under the receiver operating characteristic curve of 0.69 (95% confidence interval [CI]: 0.65–0.73) and 0.68 (95% CI: 0.64–0.72), respectively.

**Conclusion:**

In the high‐risk group, the guidelines increased the chance of detecting choledocholithiasis by approximately 10% (61.0% prevalence to 73.6 and 73.7% positive predictive value). However, statistically, the guidelines had marginal discriminative performance in a population with high stone prevalence.

## Graphical abstract

Currently, widely utilized choledocholithiasis guidelines are American Society of Gastrointestinal Endoscopy, European Society of Gastrointestinal Endoscopy, included no to low‐risk patients in their large part of the recommendation. Performance may be questionable if applied to the high‐stone‐prevalence group. We then aimed to answer this question.

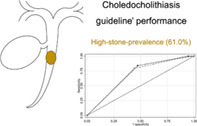

## Introduction

Choledocholithiasis (common bile duct [CBD] stones) is a condition in which stones appear in the biliary system. Choledocholithiasis is associated with many complications ranging from abdominal pain to potentially lethal cholangitis.^1^ All detected stones should be treated;^2^ however, the investigation and treatment options vary considerably.^3^ Investigations for CBD stones can involve minimally invasive methods, such as magnetic resonance cholangiopancreatography (MRCP) or endoscopic ultrasonography (EUS), with no therapeutic properties and which require other therapeutic options after CBD stones are detected, or methods such as endoscopic retrograde cholangiography (ERC). Although invasive, ERC has treatment potential and, in many institutions, this is the standard and primary treatment for CBD stones.^4^ However, ERC is associated with morbidity and, rarely, mortality;^5^ therefore, using ERC for diagnosis only should be avoided.^6^ With these challenges, many recommendations have been developed to help physicians in their decision making.^7^, ^8^, ^9^, ^10^ Currently, the American Society of Gastrointestinal Endoscopy (ASGE) and the European Society of Gastrointestinal Endoscopy (ESGE) guidelines are widely used.^2^, ^6^ The most recent ASGE (2019) guidelines are a revised version of the 2011 guidelines.^8^ Both guidelines included patients with no to low risk of having CBD stones. As the guidelines do not specify the approaches to patients with suspected CBD stones, the guidelines' accuracy may be questionable if applied to a group with high stone prevalence.^11^, ^12^ Additionally, the guidelines' predictive performances in published studies vary.^13^, ^14^, ^15^ For these reasons, our main objective in this study was to evaluate the predictive abilities for CBD stones of the ASGE 2019 and the ESGE guidelines in patients with suspected CBD stones.

## Methods

The design of the data collection was in accordance with a retrospective observational cohort study. Data for the three main reference tests, namely ERC, intraoperative cholangiography (IOC) or operative bile duct exploration, and MRCP, were reviewed from January 2019 to June 2021. The identified data were then evaluated against the eligibility criteria.

The setting was a 700‐bed tertiary hospital, and the subjects in this study comprised both local and referral cases.

The inclusion criteria were as follows:

- gallstone‐related abdominal pain with abnormal liver function tests (LFTs) or relevant abnormal imaging results (dilated bile duct or imaging‐detected CBD stones)
- gallstones with jaundice
- gallstone pancreatitis
- cholecystitis with abnormal LFTs or relevant abnormal imaging results
- cholangitis.

The diagnosis of gallstone pancreatitis, cholecystitis, and cholangitis was confirmed in accordance with standard guidelines.^16^, ^17^, ^18^

The exclusion criteria were the following:

- patients with previous biliary tract intervention (surgical or endoscopic)
- patients who had undergone cholecystectomy previously
- imaging‐confirmed morphological liver cirrhosis
- clinically suspected cancers (painless obstructive jaundice [bilirubin > 5.85 mg/dL] with anorexia and weight loss, and imaging‐confirmed bile duct dilatation without stones).^19^, ^20^ Patients in whom malignancy was initially suspected but only CBD stones were eventually confirmed were also excluded.

We collected the following clinical data: each patient's age, gender, clinical data, LFT data (aspartate aminotransferase [AST], alanine aminotransferase [ALT], alkaline phosphatase [ALP], and total bilirubin [TB]), and imaging results. Abnormal LFT results were defined as values for AST, ALT, ALP, or TB above their respective normal upper limits. TB was further categorized according to the ASGE guidelines as ≤4 mg/dL *versus* >4 mg/dL. Screening imaging methods comprised ultrasonography (US) or computed tomography (CT). In our hospital, we had a protocol for repeating LFTs before the reference tests. However, some physicians chose not to repeat the LFTs. Data were excluded if LFTs were performed more than 7 days before the reference tests. The most recent screening imaging results were used for the analysis. Recorded imaging parameters were CBD stone detection and CBD size in millimeters (mm). The CBD size was acquired from the initial reports; or, if unavailable, the size was measured from the hospital picture archiving and communication system by the participating radiologist. The bile duct measurement location was just distal to the porta hepatis or mid‐CBD. Bile duct dilatation status was not used, to avoid uncertain wording such as minimal or borderline dilatation.

The outcome—the presence of CBD stones—was recorded according to the reference tests. The tests were chosen by the attending physicians. A CBD stone was considered “positive” (detected) if it was visualized in the endoscopic or operative field in the first or a later therapeutic session. If CBD stones were not seen (such as fluoroscopic or radiologic filling defects and patients who were lost to follow‐up [FU]), images were reviewed by either two endoscopists or one of the endoscopists and the radiologist. We recorded CBD stones as “negative” (not detected) if the reference tests did not detect CBD stones during at least 5–6 months of FU to evaluate whether symptoms persisted, with normal LFT results and with or without imaging FU. Patients with less than 5–6 months of FU to evaluate whether symptoms persisted or who were lost to FU were contacted by phone to check for symptom persistence or therapy in other hospitals. “Negative” for both questions (symptom persistence and therapy in other hospitals) was required for classifying the CBD stones result as “negative.” However, we still recorded CBD stones as “negative” if the reference tests did not detect CBD stones, or the patient died or contact was lost. If a patient underwent a repeat examination with the reference tests, an FU of 5–6 months was not required. Inconclusive outcomes were excluded. All FU data were retrospectively reviewed from the hospital records data, while phone contacts were organized during the data collection process.

Data analysis was performed with the *t*‐test or the Mann–Whitney *U* test for continuous data and Fisher's exact test for categorical data. The ASGE and the ESGE guidelines' risk‐classification criteria are described in Table 1. Both guidelines classify patients into low‐, intermediate‐, and high‐risk groups,^2^, ^6^ and we categorized our patients correspondingly. The guidelines' diagnostic abilities for predicting CBD stones were then calculated. The ordinally classified data were later subjected to a logistic regression analysis to obtain the guidelines' predicted probabilities. These probabilities were used to calculate the guidelines' areas under the receiver operating characteristic curve (AUC). Missing data were managed by a complete case analysis (excluded). Statistical analysis was performed using STATA statistical software (StataCorp, College Station, TX, USA, serial number: 401709365029).

**Table 1:** The American Society of Gastrointestinal Endoscopy (ASGE) 2019 and the European Society of Gastrointestinal Endoscopy (ESGE) guidelines' risk group classification criteria

Criteria	ASGE 2019	ESGE
High risk or likelihood	Cholangitis CBD stone on US/cross‐sectional imaging Total bilirubin >4 mg/dL and dilated CBD on US/cross‐sectional imaging	Cholangitis CBD stone on US
Intermediate risk or likelihood	Abnormal LFTs Dilated CBD on US/cross‐sectional imaging Age > 55 years	Abnormal LFTs Dilated CBD on US
Low risk or likelihood	No predictors present	Normal LFT results and US findings

Dilated CBD means CBD size >6 mm.

CBD, common bile duct; LFT, liver function test; US, ultrasonography.

The study protocol was approved by the Human Research Ethics Committee of Thammasat University, Faculty of Medicine (MTU‐EC‐OO‐0‐169/64), and the Ethical Committee for Research in Human Subjects, Sawanrpacharak Hospital.

## Results

**Figure 1:**
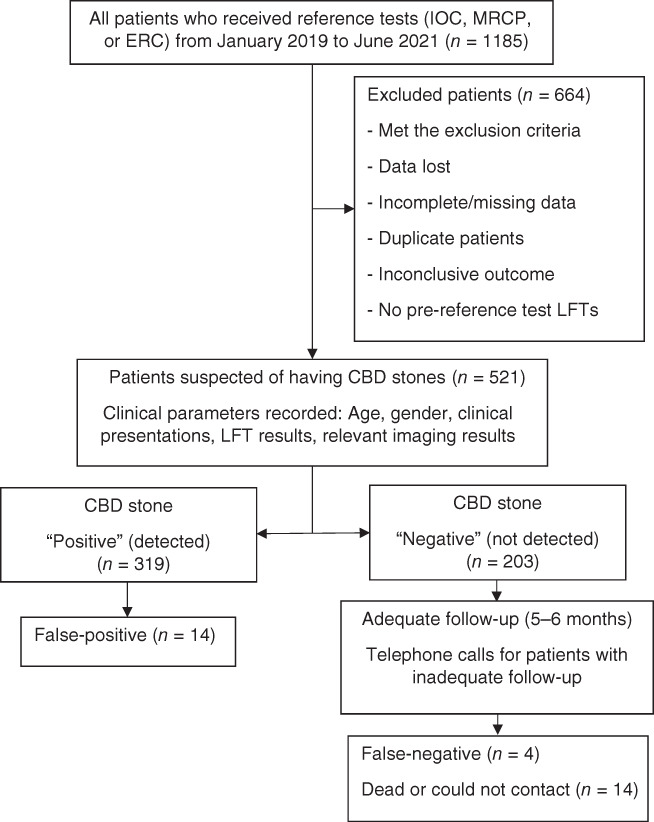
Flow diagram of the study participants. CBD, common bile duct; ERC, endoscopic retrograde cholangiography; IOC, intraoperative cholangiography; LFT, liver function test; MRCP, magnetic resonance cholangiopancreatography.

The flow diagram of the study participants is shown in Figure 1. From the data of 1185 patients who underwent investigation or treatment using the reference tests during the study period, data for 652 patients were excluded. The reasons for exclusion were compatibility with the exclusion criteria, missing data, duplicate patients, inconclusive outcomes, and having LFTs performed more than 7 days before the reference tests. Missing data for 12 patients were removed from the analysis; all were imaging results. The cause of missing data was limited ultrasonographic examination findings due to the patient's body characteristics or bowel gas status. A total of 521 patients comprised the final analyzed participants. The CBD stone prevalence was 61.0% (318 patients).

The patient characteristics are detailed in Table 2. ERC was the main reference test for 83.1% of the patients. The frequency of IOC and MRCP was equal, at 8.5%. Most patients were elderly and female, and cholangitis was the main clinical finding in 40.3% of the patients. The majority of patients had abnormal LFT results (67.4%) and dilated CBDs (80.0%). Imaging detected CBD stones in 48.8% of the patients. There were 14 (4.4%) false positives and 4 (2.0%) false negatives. Among the 14 false positives, 3 (0.94%) were benign strictures and 7 (2.2%) were cancers. However, during the analysis, we included benign strictures and cancers in the CBD stone “positive” group because both conditions usually require ERC for diagnosis or treatment. Among the CBD stone “negative” group, FU time was inadequate in 57 (28.1%) patients. We were able to contact 43 (21.2%) of these patients by telephone; 4 (2.0%) patients died, and we were unable to contact 10 (4.9%) patients.

**Table 2:** Participants' characteristics

Patient characteristic	*n* (%)
Age (years) (mean [±SD])	62.7 (17.4)
Age > 55 years	358 (68.7)
Female	323 (62.0)
Clinical presentation	
Abdominal pain	122 (23.4)
Cholecystitis	29 (5.6)
Jaundice	95 (18.2)
Pancreatitis	65 (12.5)
Cholangitis	210 (40.3)
Clinical presentations to outcome interval (days) (median [IQR])	26 (11,4)
Clinical presentations to outcome interval within 14 days	163 (31.3)
LFTs	
Abnormal LFT results	351 (67.4)
TB >4 mg/dL	85 (16.3)
Imaging results (US or CT)	
CT	126 (24.2)
CBD stone detection	255 (48.8)
CBD size (mm) (median [IQR])	10 (7.1)
Dilated CBD (>6 mm)	417 (80.0)
Reference tests	
IOC	44 (8.5)
MRCP	44 (8.5)
ERC	433 (83.1)

CBD, common bile duct; CT, computed tomography; ERC, endoscopic retrograde cholangiography; IOC, intraoperative cholangiography; IQR, interquartile range; LFT, liver function test; MRCP, magnetic resonance cholangiopancreatography; US, ultrasonography; TB, total bilirubin.

The proportion of patients with an interval between the occurrence of clinical symptoms and the reference test of within 14 days was approximately 30%. This figure reflected that most of our data were from referral cases. The median interval between screening imaging to the reference test was 8 days (interquartile range: 2, 26). CT was performed in 24.2% of the screening imaging methods.

The risk group classifications according to the guidelines are described in Table 3, and the guidelines' predictive abilities are presented in Table 4. Patients in the high‐risk categories according to both guidelines had higher proportions of CBD stones compared with patients in the intermediate‐risk groups, which had fewer stones. In our data, only 12 (2.3%) and 30 (5.8%) patients could be classified into the low‐risk group according to the ASGE and ESGE guidelines, respectively. Some patients were confirmed to have CBD stones after applying the guidelines' criteria, even in the low‐risk groups. The ESGE's low‐risk group had a high proportion of patients with CBD stones (26.7%) compared with the ASGE's low‐risk group (8.3%). Most patients were categorized as high‐risk. Our interpretation of the results focused on the high‐risk group because this is a decision‐making group. The diagnostic performances of both guidelines regarding the high‐risk groups indicated good sensitivity at approximately 80% (84.0 and 79.2% for ASGE and ESGE, respectively); however, specificity was insufficient at approximately 50% (52.7 and 55.7%, respectively). Positive predictive value (PPV) or post‐test probability can help provide more insight into data interpretation.^21^ In 61.0% of the patients' CBD stone prevalence data, the stone probability (post‐test probability) shifted to 73.6% for the ASGE guidelines and 73.7% for the ESGE guidelines after patients were categorized into the high‐risk group; that is, the chance of detecting CBD stones increased by approximately 10%. Both guidelines' high‐risk classifications had a nearly equal positive likelihood ratio (LHR+) at 1.78 (95% confidence interval [CI]: 1.52–2.07) for the ASGE guidelines and 1.79 (95% CI: 1.52–2.11) for the ESGE guidelines. The AUC, which reflected the overall discrimination ability, was 0.69 (95% CI: 0.65–0.73) for the ASGE guidelines and 0.68 (95% CI: 0.64–0.72) for the ESGE guidelines, which were comparable (*P* = 0.33).

**Table 3:** Predictors distribution according to the American Society of Gastrointestinal Endoscopy (ASGE) and the European Society of Gastrointestinal Endoscopy (ESGE) risk group criteria

Risk groups with their associated criteria	ASGE 2019		ESGE	
	CBD stone status, *n* (%)		CBD stone status, *n* (%)	
	Positive	Negative	Positive	Negative
Low risk	1 (8.3)	11 (91.7)	8 (26.7)	22 (73.3)
Intermediate risk	50 (34.3)	96 (65.7)	58 (38.9)	91 (61.1)
Abnormal LFTs	29 (33.7)	57 (66.3)	44 (41.1)	63 (58.9)
Age > 55 years	34 (35.8)	61 (64.2)	NA	
Dilated CBD on imaging	33 (37.1)	56 (62.9)	48 (43.6)	62 (56.4)
High risk	267 (73.6)	96 (26.4)	252 (73.7)	90 (26.3)
Cholangitis	155 (73.8)	55 (26.2)	155 (73.8)	55 (26.2)
CBD stone on imaging	197 (77.3)	58 (22.7)	197 (77.3)	58 (22.7)
TB > 4 mg/dL and dilated CBD on imaging	64 (86.5)	10 (13.5)	NA	

CBD, common bile duct; LFT, liver function test; NA, not available; TB, total bilirubin.

**Table 4:** Diagnostic performance of the American Society of Gastrointestinal Endoscopy (ASGE) and European Society of Gastrointestinal Endoscopy (ESGE) guidelines for common bile duct (CBD) stone prediction

	CBD stone status, *n* (%)		Diagnostic properties (95% CI)				
Guidelines classification	Positive	Negative	Sensitivity	Specificity	PPV	LHR+	AUC
ASGE 2019							
Low risk	1 (0.3)	11 (5.4)	0.3 (0.0–1.7)	94.6 (90.5–97.3)	8.3 (0.2–38.5)	0.06 (0.01–0.44)	0.69 (0.65–0.73)
Intermediate risk	50 (15.7)	96 (47.3)	15.7 (11.9–20.2)	52.7 (45.6–59.7)	34.2 (26.6–42.5)	0.33 (0.25–0.44)	
High risk	267 (84.0)	96 (47.3)	84.0 (79.5–87.8)	52.7 (45.6–59.7)	73.6 (68.7–78.1)	1.78 (1.52–2.07)	
ESGE							
Low risk	8 (2.5)	22 (10.8)	2.5 (1.1–4.9)	89.2 (84.1–93.1)	26.7 (12.3–45.9)	0.23 (0.11–0.51)	0.68 (0.64–0.72)
Intermediate risk	58 (18.2)	91 (44.8)	18.2 (14.2–22.9)	55.2 (48.1–62.1)	38.9 (31.1–47.2)	0.41 (0.31–0.54)	
High risk	252 (79.2)	90 (44.3)	79.2 (74.4–83.6)	55.7 (48.5–62.6)	73.7 (68.7–78.3)	1.79 (1.52–2.11)	

AUC, area under the receiver operating characteristic curve; CI, confidence interval; LHR+, positive likelihood ratio; PPV, positive predictive value.

The predictive ability of the guidelines for the high‐risk groups when benign bile duct stricture and cancers were excluded from the CBD “positive” group were as follows for the ASGE guidelines: sensitivity, 84.1% (95% CI: 79.5–88.0%); specificity, 52.7% (95% CI: 45.6–59.7%); PPV, 73.0% (95% CI: 68.0–77.5%), and LHR+, 1.78 (95% CI: 1.53–2.07). For the ESGE guidelines, sensitivity, 79.2% (95% CI: 74.3–83.6%); specificity, 55.7% (95% CI: 48.5–62.6%); PPV, 73.1% (95% CI: 68.0–77.7%), and LHR+, 1.79 (95% CI: 1.52–2.11). The ASGE guidelines had an AUC of 0.69 (95% CI: 0.65–0.73), and the ESGE guidelines had an AUC of 0.68 (95% CI: 0.64–0.72). The number of participants after applying the exclusion criteria was 511, and the results for this group were almost equal to the original outcomes.

## Discussion

The diagnostic abilities of the guidelines can be interpreted by assessing LHR+ and AUC.^21^, ^22^, ^23^ After classifying patients into the high‐risk group, with LHR+ results approaching 2 for both guidelines (ASGE: 1.78 [95% CI: 1.52–2.07] and ESGE 1.79 [95% CI: 1.52–2.11]), the guidelines yielded small but important benefits for CBD stone classification.^21^ The AUC or the concordance index results agreed with the LHR+ outcomes. The AUC in our study, which was calculated from all risk groups' predicted probabilities, reflected the guidelines' overall discriminative properties. AUC ≥0.7 is considered acceptable.^23^ In this study, the guidelines' AUCs were both <0.7 (ASGE: 0.69 [95% CI: 0.65–0.73]; ESGE: 0.68 [95% CI: 0.64–0.72]), and the AUCs were essentially equivalent (*P* = 0.33). Thus, the guidelines had a marginal discrimination ability. However, to our knowledge, there is still no optimal CBD stone threshold probability to guide therapeutic options.^2^ The ASGE guidelines recommend ERC at a threshold probability of >50%.^6^ After a patient was classified as high‐risk, the probability of having stones increased from 61.0% (pre‐test probability or data prevalence) to 73.6% (post‐test probability according to the ASGE guidelines) and 73.7% (post‐test probability according to the ESGE guidelines). Thus, if physicians accept the ERC risk for CBD stone probability according to the ASGE recommendations (>50%), the guidelines are considered to have clinical benefits and change the probability of CBD stones from approximately 60–70%. Regarding the low‐risk category, the ASGE guidelines appear to have superior diagnostic properties compared with the ESGE guidelines, in our study. However, because very few patients were classified into the low‐risk group, we cannot draw conclusions regarding this group.

Because both guidelines were published recently (2019), there are only a few published studies evaluating their performances. However, Jagtap *et al*. reported the guidelines' excellent performance, describing high levels for all diagnostic parameters, namely remarkable LHR+ values (ASGE: 23.8 [95% CI: 16.0–35.5] and ESGE: 71.1 [95% CI: 35.6–142.2]) and exceptional AUC values (ASGE: 0.86 [95% CI: 0.83–0.88] and ESGE: 0.87 [95% CI: 0.84–0.89]).^14^ These results contrast with the results of other studies and with our results.^13^, ^15^ The potential reason for these differences is presumably that the CBD stone prevalence in Jagtap *et al*.'s study was 26.5% compared with the >60% prevalence in other studies.^13^, ^14^, ^15^ Studies with high CBD stone prevalence evaluating the ASGE 2011 guidelines' performance reported similar results.^13^, ^24^, ^25^, ^26^ These findings could indirectly mean that the guidelines are accurate if CBD stone prevalence is low but they are not likely to be accurate if the risk is high. We have summarized previous studies' analyses of the predictive ability of the guidelines' high‐risk classification according to the patients' CBD stone prevalence in Table 5. We also calculated and presented non‐reported statistical values (most were AUCs) from the studies' provided data if the relevant statistical data were not available. Importantly, all of the published studies presented in Table 5 were retrospective studies (subject to some biases), and they varied in reference test methods for CBD stone confirmation.

**Table 5:** Studies evaluating the high‐risk group predictive ability of the American Society of Gastrointestinal Endoscopy (ASGE) 2011, ASGE 2019, and European Society of Gastrointestinal Endoscopy (ESGE) guidelines for common bile duct (CBD) stones

	*n* (%)							
Study (ref number) (*n* = number of patients)	CBD stone prevalence	High‐risk prevalence	Stones in high‐risk patients	Sens.	Spec.	PPV	LHR+ (95% CI)	AUC (95% CI)
ASGE 2011								
Adams et al.^27^ (*n* = 498)	210 (42.2)	179 (35.9)	99 (55.3)	47.4	73.0	55.3	1.70 (1.34–2.15)	0.60 (0.55–0.64)
He et al.^28^ (*n* = 2724)	1076 (39.5)	1171 (43.0)	750 (64.1)	69.7	74.5	64.0	2.73 (2.49–2.99)	0.72 (0.70–0.74)
Jacob et al.^13^ (*n* = 267)	192 (71.9)	165 (61.8)	131 (79.4)	68.2	54.7	79.4	1.51 (1.15–1.96)	0.61 (0.55–0.68)
Kuzu et al.^26^ (*n* = 888)	704 (79.3)	550 (61.9)	477 (86.7)	67.8	60.3	86.7	1.71 (1.42–2.06)	0.64 (0.60–0.68)
Magalhaes et al.^24^ (*n* = 268)	179 (66.8)	193 (72.0)	154 (79.8)	86.0	56.2	79.8	1.96 (1.54–2.50)	0.71 (0.65–0.77)
Rubin et al.^25^ (*n* = 521)	293 (56.2)	264 (50.7)	189 (71.6)	64.5	67.1	71.6	1.96 (1.60–2.40)	0.66 (0.62–0.70)
ASGE 2019								
Jacob et al.^13^ (*n* = 267)	192 (71.9)	86 (32.2)	71 (82.6)	37.0	80.0	82.6	1.85 (1.13–3.01)	0.58 (0.53–0.64)
Jagtap et al.^14^ (*n* = 1042)	276 (26.5)	230 (22.1)	206 (89.6)	74.6	96.9	89.6	23.82 (15.97–35.53)	0.86 (0.83–0.88)
Lei et al.^15^ (*n* = 879)	622 (70.8)	472 (53.7)	370 (78.4)	59.5	60.3	78.4	1.50 (1.27–1.77)	0.60 (0.56–0.63)
Current study (*n* = 521)	318 (61.0)	363 (69.7)	267 (73.6)	84.0	52.7	73.6	1.78 (1.52–2.07)	0.68^a^ (0.64–0.72)
ESGE								
Jagtap et al.^14^ (*n* = 1042)	276 (26.5)	213 (20.4)	205 (96.2)	74.3	99.0	96.2	71.12 (35.57–142.19)	0.87 (0.84–0.89)
Lei et al.^15^ (*n* = 879)	622 (70.8)	338 (38.5)	288 (85.2)	46.3	80.5	85.2	2.38 (1.83–3.10)	0.63 (0.60–0.67)
Current study (*n* = 521)	318 (61.0)	342 (65.6)	252 (73.7)	79.2	55.7	73.7	1.79 (1.52–2.11)	0.67^a^ (0.63–0.72)

^a^ AUC specific to the high‐risk group.

AUC, area under the receiver operating characteristic curve; CI, confidence interval; LHR+, positive likelihood ratio; PPV, positive predictive value; Sens., sensitivity; Spec., specificity.

Although the statistical analysis in this study was designed for the diagnostic tests, the guidelines are not the diagnostic modalities. Many diagnostic or therapeutic options are followed after risk group classification based on the availability of local expertise or resources. Our study's aim was not to decrease the guidelines' credibility. However, a considerable number of patients categorized as high‐risk had no CBD stones (26.3–26.4%). Therefore, we encourage physicians to use their available resources as efficiently as possible to limit patient morbidity from CBD stone investigations, particularly in facilities with high stone prevalence, such as referral hospitals. For example, EUS has gained more utility, with diagnostic accuracy comparable to or better than MRCP.^29^ The noteworthy aspect is that EUS and ERC in the same setting can help avoid unnecessary ERC,^30^ which potentially benefits high‐risk patients.

There were limitations in our study. First, we could not include all patients with suspected CBD stone in our data because we reviewed the reference test data rather than the patients' clinical diagnoses. Some patients could have had minimal LFT or imaging abnormalities as diagnosed by the attending physician who chose to observe the patients. The guidelines' accuracy might have been higher if this group of patients had been included. However, because the validity of the outcome was more important, removing the questionable results from the data might have been more appropriate. Second, this was a retrospective study, and we managed missing data by excluding these patients, which might have led to information loss. Third, our reference tests did not include all CBD stone confirmation tests. For example, EUS was not available in our hospital during the study period. Lacking certain reference tests and missing some patients could have affected the guidelines' performance.^31^ Fourth, US is traditionally considered a screening method for CBD stones. However, CT, at least in our country, is increasingly accessible, especially when radiologists are not available. Conducting a CBD stone study with US as a pure screening test is increasingly difficult and nearly impossible. The ASGE guidelines also added cross‐sectional imaging as a screening method for CBD stone.^6^ Although this issue may be a limitation, CT was performed in approximately 25% of basic screenings, which would not have greatly affected the outcomes. Finally, we included patients with malignancy and benign bile duct stricture as “positive” cases of CBD stones because of the potential benefits of ERC. A similar situation could occur in clinical practice. However, we also calculated the guidelines' diagnostic parameters after excluding patients with malignancy and benign bile duct stricture, and the more homogenous group gave almost the same results.

In conclusion, with a CBD stone prevalence of approximately 60%, the ASGE and ESGE guidelines had statistically marginal classification or discrimination ability. However, clinically, the guidelines could assist in obtaining accurate classification in approximately 10% of cases. Nevertheless, roughly one‐fourth of patients might suffer risks from diagnostic ERC if the high‐risk group undergoes this procedure.

## Informed consent

The need to obtain patient consent was waived because of the retrospective study design and the use of de‐identified data.
